# Characterization of MaltOBP1, a Minus-C Odorant-Binding Protein, From the Japanese Pine Sawyer Beetle, *Monochamus alternatus* Hope (Coleoptera: Cerambycidae)

**DOI:** 10.3389/fphys.2020.00212

**Published:** 2020-04-01

**Authors:** Fangmei Zhang, Austin Merchant, Zhibin Zhao, Yunhui Zhang, Jing Zhang, Qingwen Zhang, Qinghua Wang, Xuguo Zhou, Xiangrui Li

**Affiliations:** ^1^Henan Provincial South Henan Crop Pest Green Prevention and Control Academician Workstation, Xinyang Agriculture and Forestry University, Xinyang, China; ^2^State Key Laboratory for Biology of Plant Diseases and Insect Pests, Institute of Plant Protection, Chinese Academy of Agricultural Sciences, Beijing, China; ^3^Department of Entomology, University of Kentucky, Lexington, KY, United States; ^4^Key Laboratory of Biorheological Science and Technology, Ministry of Education, College of Bioengineering, Chongqing University, Chongqing, China; ^5^Department of Entomology, China Agricultural University, Beijing, China; ^6^The Key Laboratory of Forest Protection, State Forestry Administration of China, Research Institute of Forest Ecology, Environment and Protection, Chinese Academy of Forestry, Beijing, China

**Keywords:** *Monochamus alternatus*, olfactory, odorant binding protein, fluorescence binding assay, plant volatile

## Abstract

Insect Odorant-Binding Proteins (OBPs) play crucial roles in the discrimination, binding and transportation of odorants. Herein, the full-length cDNA sequence of Minus-C OBP1 (MaltOBP1) from the Japanese pine sawyer beetle, *Monochamus alternatus*, was cloned by 3′ and 5′ RACE-PCR and analyzed. The results showed that MaltOBP1 contains a 435 bp open reading frame (ORF) that encodes 144 amino acids, including a 21-amino acid signal peptide at the N-terminus. The matured MaltOBP1 protein possesses a predicted molecular weight of about 14 kDa and consists of six α-helices, creating an open binding pocket, and two disulfide bridges. Immunoblotting results showed that MaltOBP1 was most highly expressed in antennae in both sexes, followed by wings and legs. Fluorescence assays demonstrated that MaltOBP1 protein exhibited high binding affinity with (R)-(+)-α-pinene, (−)-β-pinene, *trans*-caryophyllene, (R)-(+)-limonene and (–)-verbenone, which are the main volatile compounds of the pine tree. Our combined results suggest that MaltOBP1 plays a role in host seeking behavior in *M. alternatus*.

## Introduction

Insects rely on sophisticated olfaction to detect semiochemicals at critical stages of the life cycle, subsequently undertaking a series of corresponding behavioral responses, such as mating, oviposition, foraging, host seeking and predator avoidance (Zhang et al., 2015; Elgar et al., 2018). Many proteins have been found to be involved in odorant reception in major chemosensory organs (Leal, 2013). Among these proteins, odorant-binding proteins (OBPs) are commonly accepted to act as the first step in olfaction in insects (Brito et al., 2016; Pelosi et al., 2018).

In general, insect OBPs are small globulins consisting of 150–250 amino acids, and possess a common fold of six α-helical domains that form a conserved pocket, which is further stabilized by three interlocking disulfide bridges between six highly conserved cysteine residues, which are crucial to stabilizing the three-dimensional structure of the binding cavity (Northey et al., 2016; Venthur and Zhou, 2018). An extremely large number of OBP homologs have been isolated and cloned from several insect orders, including Lepidoptera, Diptera, Hemiptera, Coleoptera, Homoptera, and Orthoptera (Pelosi et al., 2018; Venthur and Zhou, 2018). Insect OBPs belong to a multigene family, which has been categorized into four distinct types according to the number of conserved cysteine residues present. These types are “Classic OBPs” (with six conserved cysteines), “Minus-C OBPs” (with four conserved cysteines), “Plus-C OBPs” (with eight conserved cysteines), and “Atypical OBPs” (with more than eight conserved cysteines) (Venthur et al., 2014; Brito et al., 2016).

Studies involving OBP identification (Li L. et al., 2017; Zhao et al., 2018), protein structural analysis (Northey et al., 2016; Falchetto et al., 2019), immunocytochemistry in specific olfactory sensilla (Ma L. et al., 2018; Zhang et al., 2018), ligand binding properties (Sun et al., 2017, 2018), and *in vivo* RNA interference (Yin et al., 2018; He et al., 2019) have demonstrated that insect OBPs play crucial roles in odorant discrimination, binding, and transportation. These proteins carry lipophilic odorants to the olfactory receptor cells in the sensillar lymph surrounding the sensory dendrite (Pelosi et al., 2014, 2018).

The expression patterns of OBPs among different tissue types can offer valuable information for determining their functions. Most insect OBPs are highly expressed in antennae, which indicates that OBPs play an important role in chemoreception. However, recent studies have found expression of OBPs in non-sensory organs, such as in *Locusta migratoria* and *Schistocerca gregaria* OBP8 with considerable expression in palps (Li et al., 2018; Pregitzer et al., 2019), *Rhynchophorus ferrugineus* RferOBP7 and RferOBP11 with head-biased and thorax-biased expression in females and males, respectively (Yan et al., 2016), and *Rhodnius prolixus* RproOBP with overexpression in the gut (Ribeiro et al., 2014). These broad and diverse expression patterns indicate that OBPs are more complicated than previously imagined, with possible roles beyond chemoreception.

The Japanese pine sawyer beetle, *Monochamus alternatus* Hope (Coleoptera: Cerambycidae), is a major quarantine pest of pine forests in China, causing serious ecological damage and economic losses. Since 1985, half of all pine trees, *Pinus massoniana*, in China have been wiped out by this pest, and the combined costs of damage and management exceed $4 billion USD annually (Shi et al., 2008; Xu et al., 2008). Although other life stages remain within a single host tree, adults expose themselves to open air to find new pine trees for supplementary nutrition and to lay eggs. Adult beetles also act as vectors for the pine wood nematode (PWN) *Bursaphelenchus xylophilus* (Steiner and Buhrer) Nickle (Nematoda: Aphelenchoididae), the causal agent of pine wilt disease (Alves et al., 2016; Mckenna et al., 2016; Ma T. et al., 2018). Thus, the adult stage of the Japanese pine sawyer beetle is the most important stage for the management of pinewood nematodes.

*Monochamus alternatus* possesses a complex olfactory system, and different populations of *M. alternatus* might use different host volatiles as kairomones with different host plants and forest conditions (Teale et al., 2011). Monoterpenes (α- and β-pinene), which are produced by pine trees (Zhao et al., 2007), were identified as the primary attractants for adult Asian longhorned beetles. The synergistic effects of other host plant volatiles, such as 3-carene, β-caryophyllene, limonene, myrcene, and β-pinene have previously been confimed in *Monochamus* species (Lee et al., 2017, 2018). In recent years, pine volatiles and sex pheromones have been used to develop various types of attractants that are effective in controlling *M. alternatus* (Fan et al., 2010; Lee et al., 2018; Ma T. et al., 2018). Furthermore, previous studies have demonstrated that these host-derived volatile compounds could also enhance the attractiveness of the *Monochamus* aggregation-sex pheromone (Lee et al., 2017).

Up to date, only a few Minus-C OBPs have been documented in *M. alternatus*. MaltOBP3 and MaltOBP5 bound host-plant volatiles and implicated a role in host-plant selection (Gao and Wang, 2014), while expression profiles of MaltOBP2 and MaltOBP6 projected their roles in olfaction, taste, and chemosensation (Qian et al., 2015).

The overall goal of this research is to study the molecular basis of host-seeking behavior in *M. alternatus*. Based on our preliminary research, we hypothesized that MaltOBP1, a Minus-C OBP, contributes to the host plant localization. To test this hypothesis, we carried out the following objectives: (1) molecular cloning and characterization of MaltOBP1, (2) Spatial profiling of MaltOBP1 protein expression among different tissues, and (3) assessing the binding affinity of recombinant MaltOBP1, in the context of host-seeking behavior.

## Materials and Methods

### *Monochamus alternatus* Colony Maintenance

*Monochamus alternatus* colonies were originally collected from dead pine trees in Fuyang, Hangzhou, Zhejiang Province, China (119.95°N, 30.05°S) and maintained at the Research Institute of Forest Ecology, Environment, and Protection, Chinese Academy of Forestry, Beijing 100091, China. Individual larvae were placed in 20 mL plastic insectary cups (φ2.5 cm) and fed on proprietary artificial diet until pupation. The larvae were kept in a growth chamber at 24 ± 1°C with a relative humidity of 60 ± 10% and a photoperiod of 16 L: 8 D. After emergence, *M. alternatus* adults were provisioned with branches and short-cut wood from fresh pine, *Pinus massoniana*.

### Molecular Cloning and Characterization of MaltOBP1

#### Protein Extraction and Sequencing

At the 4th day post-emergence, *M. alternatus* tissue from both sexes (five males and five females) was dissected on ice and processed immediately for protein extraction. Tissues types used included antennae, head (without antennae), thorax, abdomen, leg, and wing. Samples were homogenized into powder in a mortar filled with liquid nitrogen, then transferred into 1.5 mL tubes containing 20 mM Tris–HCl, pH 7.4, and then centrifuged twice at 12,500 × *g* for 10 min at 4°C. The supernatants were evaporated under a vacuum (EZ550Q, Ultralow Freezer System, FTS Systems Inc., Stone Ridge, NY, United States). Protein samples were processed for PAGE analysis at 100 V (constant voltage) and 4°C, then electroblotted onto PVDF membranes (0.2 μm, Millipore, Billerica, MA, United States) for 1.5 h at 40 mA (constant current) and 4°C. The amount of soluble protein loaded in each lane was approximately 35 μg for each tissue extract. Bands of interest were carefully excised and processed for sequencing through Edman degradation (the Procise^®^ cLC Protein Sequencing System, Applied Biosystems, Shanghai GeneCore BioTechnologies Co., Ltd., Shanghai, China).

#### RNA Extraction, cDNA Synthesis, and Molecular Cloning

Total RNA was extracted from a male antenna with TRIzol reagent (Invitrogen, United States) according to the recommended protocol. First strand cDNA was synthesized using a 3′-full RACE core set (Takara, Dalian, China) according to the manufacturer’s protocol. Degenerate sense primers were designed on the basis of the protein N-terminal sequences (Supplementary Table S1) and synthesized by Bio Basic Inc. (Shanghai, China). Reactions were performed on a Biometra TGradient PCR thermocycler: 94°C for 8 min, 5 cycles of 94°C for 30 s, 37°C for 1 min, 2 min ramp, 72°C for 1 min, followed by 30 cycles of 94°C for 30 s, 43°C for 1 min, 2 min ramp, 72°C for 1 min, and a final extension step of 10 min at 72°C. 5′ cDNA sequences were obtained using a FirstChoice^®^ RLM-RACE Kit (Ambion, Foster City, CA, United States) following the recommended protocol. Gene specific antisense primers were designed from the results of the 3′ RACE procedure (Supplementary Table S1). Reaction conditions for PCR were: 94°C for 4 min, 30 cycle of 94°C for 30 s, 60°C for 45 s, 72°C for 45 s, and a final extension step of 10 min at 72°C. The purified PCR products were ligated into a pGM-T vector and positive clones were sequenced.

#### Structural Analysis

Open reading frame (ORF), protein translation, isoelectric point (pI), and theoretical molecular weight (Mw) were calculated using Expasy^1^. Putative N-terminus signal peptides and the mature protein sequence were predicted by SignalP 4.1^2^. Similarity searches were performed with NCBI-BLAST^3^. DNAMAN v5.2.2 was used for multiple analyses of homologous sequences. Amino acid sequences were aligned using ClustalW. The phylogenetic tree was constructed using the Maximum Likelihood method with the Jones-Taylor-Thornton (JTT) model, as implemented in MEGAX software. Node support was assessed using a bootstrap procedure with 1000 replicates (Tamura et al., 2011).

The three-dimensional structure of MaltOBP1 was simulated by an online protein structure homology-modeling server, SWISS-MODEL^4^. PyMOL-v1.3r1 (Delano Scientific LLC^5^) was used for molecular visualization and labeling of structural features, such as α-helices and disulfide bridges. The protein template used in SWISS-MODEL was AgamOBP1 (PDB ID: 2ERB), an odorant binding protein with a resolved crystal structure from *Anopheles gambiae* (Wogulis et al., 2006).

### Spatial Profiling of MaltOBP1 Protein Expression Among Different Tissues

#### Recombinant Protein Expression and Purification

The recombinant *MaltOBP1* gene (without signal peptide) was inserted into *Escherichia coli* expression vector pET32a (Novagen, Madison, WI, United States) with N-terminus 6 × His tag and subsequent TEV cleavage site using recombinant PCR. The pET32a-His_TEV_OBP1 plasmids were used to transform *E. coli* BL21 (DE3) cells and the positive clones were grown in LB broth and then induced with 1 mM Isopropyl β-D-1-thiogalactopyranoside (IPTG) for about 16 h at 16°C. Bacterial cells were collected through centrifugation at 6000 rpm for 10 min at 4°C. Cell pellets were resuspended in a lysis buffer consisting of 20 mM Tris–HCl pH 7.5, 500 mM NaCl and 5% (v/v) glycerol and lysed by passing through microfluidics two times. The lysate was then centrifuged at 20,000 × *g* for 60 min. The supernatant was then processed in a Ni-NTA column (Qiagen) to obtain the purified His-tagged protein. The desired protein was eluted by gel filtration (Hiload superdex 75 16/60) to obtain the homogeneous protein with a final buffer of 20 mM Tris–HCl pH 7.5, 150 mM NaCl. The purity of the resulting protein was detected by SDS-PAGE analysis and protein molecular weight was estimated by LC-MS. The purified protein was concentrated to 2 mg/mL and stored at −80°C until use.

#### Antisera Preparation

Polyclonal antiserum against recombinant MaltOBP1 was obtained by consecutive 4-time injection (200 μg, once 14 days after primary immunization to conduct enhancement immunization, and then three times, once every 10 days) of purified recombinant proteins into New Zealand white rabbits. Animals were bled 10 days after the last injection and the serum supernatant was obtained through centrifugation at 4°C, 12,000 rpm for 15 min. The ELISA method (Enzyme-Linked ImmunoSorbant Assay) was used to assess the reactivity of antiserum.

#### Immunoblot Analysis

Total proteins from different tissues of male and female beetles, including antennae, head (without antennae), thorax, abdomen, leg, and wing tissues, were extracted and standardized to 7.5 μg per sample. After electrophoretic separation, protein bands were *trans-*ferred from a 15% SDS-PAGE to a nitrocellulose membrane (0.2 μm, Millipore, United States), as described by Kyhse-Andersen (1984). After treatment with 0.2% non-fat dry milk and 0.05% Tween-20 in PBS overnight, the nitrocellulose membrane was then incubated with the primary antiserum obtained previously at a dilution of 1:5000 based on the ELASA result in Supplementary Figure S1. Goat anti-rabbit IgG- horseradish peroxi-dase conjugate (diluted by 1:1000; Fermentas, MD, United States) was used as the secondary antibody. Immunoreactions were visualized by adding 5-bromo-4-chloro-3- indolyl-phosphate and 4-chloro-1-naphthol (Promega, WI, United States).

### Assessing the Binding Affinity of Recombinant MaltOBP1

#### Competitive Ligand-Binding Assay

Ligand-binding assays were performed on a HORIBA FluoroMax^®^-4 Fluorescence spectrophotometer (HORIBA Scientific, United States) with a 1 cm light path quartz cuvette and 5 nm slits for both excitation and emission. The purified MaltOBP1 protein, served in the 1 cm light path quartz cuvette, was titrated with 1 nM 1-N-phenyl naphtylamine (1-NPN, Sigma, Riedstr, Steinheim, Germany) solution in methanol. The fluorescence intensity at the maximum emission wavelength of about 337 nm was linearized using the Scatchard equation (Sideris et al., 1992), and then binding affinity for MaltOBP1/1-NPN was calculated.

A total of 15 candidate volatiles were selected as test ligands, which are listed in Table 1. To assess the binding affinities of ligands to MaltOBP1, a mixture of 1-NPN and MaltOBP1 protein was titrated with 1 μM of each ligand dissolved in methanol. IC_50_ was obtained by data linearization, and the dissociation constant (*K*_i_) was calculated following the equation: *K*_i_ = [IC_50_]/(1 + [1-NPN]/*K*_1__–NPN_), where [1-NPN] is the free concentration of 1-NPN and *K*_1__–NPN_ is the dissociation constant of the protein/1-NPN complex (Campanacci et al., 2001), which was calculated from the binding curve using GraphPad Prism 5.01 (GraphPad Software, Inc.).

**TABLE 1:** Binding affinity of selected compounds to the recombinant MaltOBP1.

**Ligands**	**Formula**	**CAS No#**	**Purity (%)**	**IC_50_(μM)**	***K*_i_ (μM)**
(+)-α-Pinene	C_10_H_16_	2437-95-8	≥99.00	31.49	18.67
β- Pinene	C_10_H_16_	127-91-3	≥99.00	39.32	23.30
Comphene	C_10_H_16_	565-00-4	≥98.00	—	—
myrcene	C_10_H_16_	123-35-3	≥98.00	—	—
β-Phellandrene	C_10_H_16_	555-10-2	≥98.00	—	—
(−)-*trans*-caryophyllene	C_15_H_24_	87-44-5	≥98.00	7.01	4.15
(−)-Isolongifolene	C_15_H_24_	1135-66-6	≥98.00	—	—
Cinene	C_10_H_16_	138-86-3	≥98.00	—	—
(*R*)-(+)-limonene	C_10_H_16_	5989-27-5	≥98.00	21.69	12.85
(*S*)-(+)-limonene	C_10_H_16_	5989-54-8	≥98.00		
(+)-3-carene	C_10_H_16_	20296-50-8	≥98.00	—	—
(+)-Terpinol	C_10_H_18_O	2451-01-6	≥98.00	—	—
myrcene	C_10_H_16_	123-35-3	≥98.00	—	—
(−) -verbenone	C_10_H_14_O	1196-01-6	≥99.00	63.09	37.38
Ethanol	C_2_H_5_OH	64-17-5	≥99.50	—	—

*All selected compounds are from Sigma-Aldrich.“—”indicates that the *K*_*i*_ exceeded 50 μM. Low *K*_*i*_ values mean high binding affinity for protein and plant volatiles.*

## Results

### Molecular Cloning and Characterization of MaltOBP1

#### Molecular Cloning and Sequence Analysis

**FIGURE 1:**
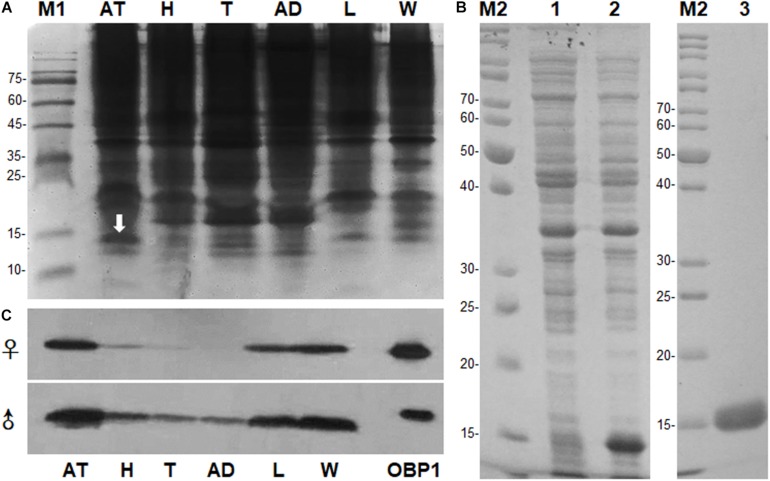
Sex- and tissue-expression profile of MaltOBP1. **(A)** Electrophoretic analysis of soluble proteins from *Monochamus alternatus* under 15% native PAGE. M1: Protein molecular weight marker. **(B)** Recombinant protein analyzed by SDS-PAGE. M2: Protein molecular weight marker; Lane 1: Non-induced pET32a-MaltOBP1 in *Escherichia coli*; Lane 2: Expressed protein pET32a-MaltOBP1-His after induction by IPTG; Lane 3: pET32a-MaltOBP1 protein purified through Ni-NTA column; **(C)** Western blot analysis of MaltOBP1 expression in total protein extracts of male and female adults of *M. alternatus*. AT, antennae; H, head; T, thorax; AD, abdomen; L, leg; W, wing; OBP1, Recombinant MaltOBP1.

The PAGE results revealed the occurrence of a specific band with molecular weight (Mw) approximately 15 kDa in antennal extracts, which we hypothesized to be an OBP (Figure 1A). The band of interest on native PAGE was cut out after electroblotting with PVDF membranes, and the resultant N-terminal sequence was IKDESELVDENGELI. Full-length sequences were obtained from 3′ and 5′ RACE-PCR. BLAST analysis showed that the gene belonged to the OBP family. We named it MaltOBP1 and deposited it in GenBank under the accession number EF593044.1. The MaltOBP1 cDNA sequence contains a 435-bp ORF and encodes a 144-amino acid protein with a 21-residue hydrophobic signal peptide (Supplementary Figure S2). The predicted molecular weight (Mw) for MaltOBP1, with and without a signal peptide, is 16.44 kDa and 14.09 kDa, respectively, with an isoelectric point (pI) of 4.74 and 4.57. The total number of negatively charged residues (Asp + Glu) is 26, and the number of positively charged residues (Arg + Lys) is 18.

**FIGURE 2:**
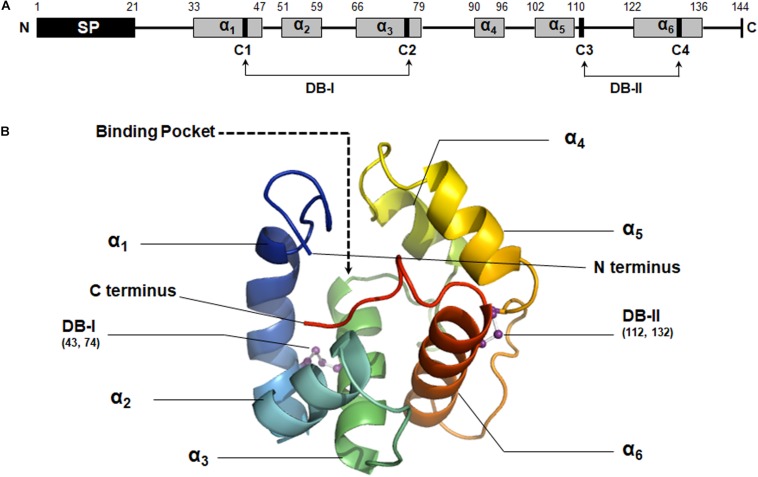
Structural analysis of MaltOBP1. **(A)** The secondary structure of MaltOBP1. **(B)** The 3D structure of MaltOBP1. The six α-helices are labeled as α_1_–α_6_. DB-I, Disulfide bridge-I; DB-II, Disulfide bridge-II.

The secondary structure of MaltOBP1 consists of six α-helices: α1 (residues 33 to 47), α2 (residues 51 to 59), α3 (residues 66 to 79), α4 (residues 90 to 96), α5 (residues 102 to 110), and α6 (residues 122 to 136). A total of two disulfide bridges (DB-I and DB-II) were observed between Cys43 and Cys74, and Cys112 and Cys132 (Figure 2A).

Using homology modeling from Swiss-Model Workspace, a three-dimensional (3D) model of MaltOBP1 was generated based on AgamOBP1 (Wogulis et al., 2006), a protein template from *Anopheles gambiae* (PDB ID: 2ERB). The sequence similarity is 34.48%. A Ramachandran plot revealed that 97.41% of the residues were in the allowed region, and 2.59% were located near the marginal region and none was in the disallowed region, indicating that the overall stereochemical quality of the predicted tertiary structure is reliable and acceptable. As a α-helix-rich protein, the predicted MaltOBP1 tertiary structure contains an internal cavity, representing a binding pocket. The N-terminus and C-terminus are medium long but do not form a helix (Figure 2B).

#### Phylogenetic Analysis

The results of multiple amino acid sequence alignment of MaltOBP1 with 12 Minus-C OBPs are shown in Supplementary Figure S3. These OBPs possess the four conserved cysteine residues that are typical of a Minus-C OBP. Supplementary Figure S4 shows a phylogenetic tree of MaltOBP1 and 91 OBPs that have been published previously from *M. alternatus* (24 OBPs), sibling species *Anoplophora glabripennis* (16 OBPs), and two other coleopteran insects, *Leptinotarsa decemlineata* (26 OBPs) and *Tribolium castaneum* (25 OBPs). The closest relatives of MaltOBP1 are AglaOBP8, MaltOBP22, AglaOBP9, MalOBP23, AglaOBP1, MaltOBP12, LdecOBP8, LdecOBP11, LdecOBP6, LdecOBP7, and LdecOBP3. These OBPs are clustered into the same clade, which also belongs to Minus-C OBPs.

### Spatial Profiling of MaltOBP1 Protein Expression Among Different Tissues

SDS-PAGE analysis showed a single protein band of size slightly greater than 15 kDa (Figure 1B). The molecular weight of recombinant MaltOBP1 protein with a His-TEV-tag (∼2 kDa) was then analyzed by LC-MS. The major peak was at 16.05 kDa (Supplementary Figure S5), which is consist with the predicted molecular weight of MaltOBP1 (∼14 kDa) plus a TEV-tag (∼2 kDa). Western blot results revealed that MaltOBP1 was ubiquitously expressed throughout all tissue types, including antennae, wings, and legs of adult males and females (Figure 1C).

### Assessing the Binding Affinity of Recombinant MaltOBP1

**FIGURE 3:**
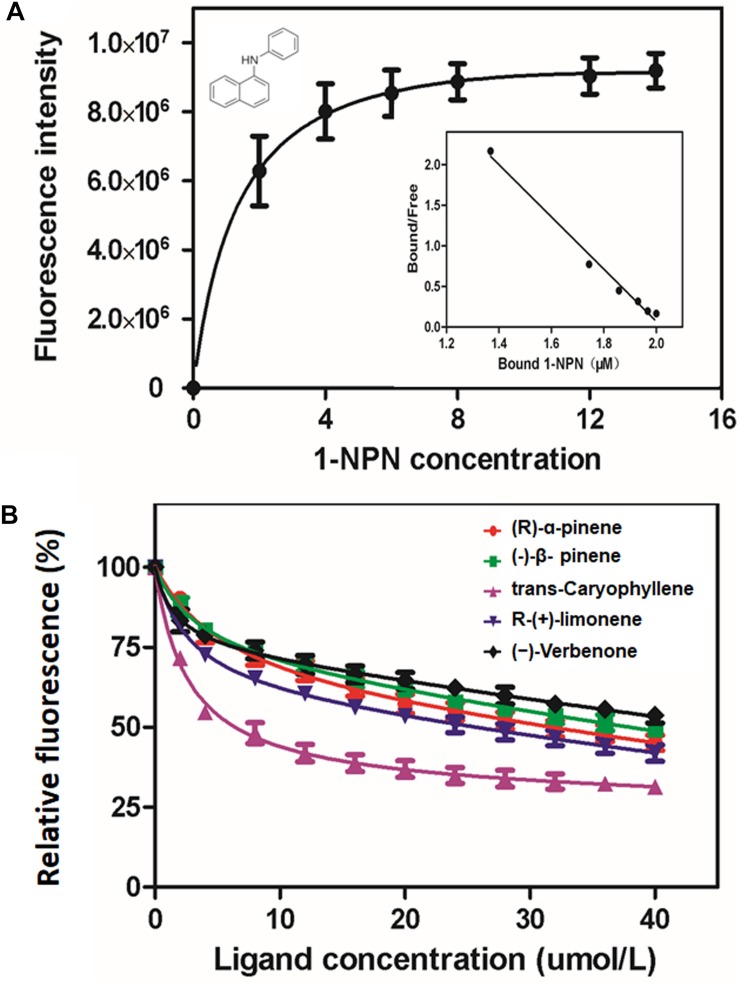
Binding affinity of MaltOBP1. **(A)** Binding curve and relative Scatchard plot of MaltOBP1 and 1-NPN. **(B)** Competitive binding curves of selected volatile host plant compounds to MaltOBP1.

The binding plots of MaltOBP1 suggested that the binding of 1-NPN was saturable and consistent with a single population of binding sites, with no apparent allosteric effect. The dissociation constant of MaltOBP1/1-NPN was measured as 3.86 μM (Figure 3A). Competitive binding curves are shown in Figure 3B. Table 1 lists IC_50_ values and dissociation constants (*K*_i_) of all examined ligands. These results suggest that MaltOBP1 has strong binding affinity to five compounds, including (+)-α-pinene (18.67 μM), β-pinene (23.30 μM), (−)-*trans*-caryophyllene (4.15 μM), (R)-(+)-limonene (12.85 μM) and (–)-verbenone (37.38 μM). Among these five compounds, (−)-*trans*-caryophyllene exhibited the highest affinity to MaltOBP1, with a *K*_i_ value of 4.15 μM.

## Discussion

### Structural Features of MaltOBP1

MaltOBP1, a secretable protein, has an N-terminus domain containing a 21-amino acid signal peptide. Multiple sequence alignment shows that MaltOBP1 shares high sequence similarity with other Minus-C OBPs, which possess four conserved cysteine residues that follow a common pattern: C_1_-X_30_-C_2_-X_37_-C_3_-X_19_-C_4_ (where X represents any amino acid) (Zhou, 2010). Previous studies of the origins and evolutionary histories of chemosensory systems suggest that Minus-C OBPs might be the ancestors of Classic-C OBPs (Vieira and Rozas, 2011; Li et al., 2013). Compared to Classic-C OBPs, the loss of one disulfide bridge in Minus-C OBPs might also have functional relevance.

Phylogenetic analysis placed MaltOBP1 into the same branch of Minus-C OBPs with other OBPs from closely related coleopteran species, *A. glabripennis* and *L. decemlineata*. To date, there has been no report on the function of these minus-C OBPs in beetles (Wang et al., 2014; Ping et al., 2016; Zhang et al., 2019). We speculate that these genes are homologous genes that are evolutionarily conserved and have similar biological functions.

The projected MaltOBP1 tertiary structure exhibited conserved structural features, including six α-helices and an internal cavity (Sun et al., 2016; Zheng et al., 2016). MaltOBP1 possesses only two disulfide bridges and a hydrophobic binding cavity, which is similar to other Minus-C OBPs, including *Apis mellifera* AmelOBP14 (Spinelli et al., 2012), *Batocera horsfieldi* BhorOBPm2 (Zheng et al., 2016), and *Dastarcus helophoroides* DhelOBP21 (Li et al., 2015). However, the structure features of Minus-C OBPs are diverse, with a wide variety of cavity shapes and positions, solvent accessibilities, and amino acid sequences (Tsitsanou et al., 2013). The C-terminus has been shown to be an important component in binding and releasing OBPs to odorants (Sun et al., 2016). In AmelOBP14 and *Ceratitis capitata* CcapOBP22, which belong to a class of OBPs that possesses eight cysteines (Plus-C OBPs), the C-terminus segment forms an external seventh helix at the interface between the protein exterior and the internal cavity (Spinelli et al., 2012; Falchetto et al., 2019). However, *D. helophoroides* DhelOBP21, with a short C-terminus, possesses five helices, unlike other insect OBPs (Li et al., 2015). MaltOBP1 has a medium length C-terminus, which is located near the opening of the binding site, instead of an exposed C-terminus helix. Two Minus-C OBPs with a medium sized C-terminus, HarmOBP17 and HarmOBP18, displayed a higher binding affinity at pH5.0 than pH7.4, suggesting that increase in acidity caused the C-terminal loop to open, and exposed the binding tunnel to solvent (Li et al., 2013). Given that, we speculate that different Minus-C OBPs could display different mechanisms for binding and releasing of odorants. Additionally, the plasticity and volume of the binding pocket might produce differences in ligand selection. AmelOBP14 has a closed core in contrast to the open pocket observed in MaltOBP1 and DhelOBP21, which suggests that elongated compounds may be more suitable for the latter group (Zheng et al., 2016).

### Tissue-Specificity of MaltOBP1

Analyzing expression patterns of OBPs could better help us to understand the functions of these proteins. Many studies have shown that most insect OBPs are mainly expressed in the antennae of both sexes, demonstrating that they might be involved in olfactory functions (Li L. et al., 2017; Zhang et al., 2017; He et al., 2019). Our western blot experiments show that MaltOBP1 is predominantly expressed in the antennae, legs and wings of both sexes. A similar expression pattern has been observed in *Helicoverpa armigera* HarmOBP18 (Li et al., 2013) and *Spodoptera exigua* SexiOBP9 (Liu N. Y. et al., 2015). Elevated expression in non-olfactory regions, i.e., leg and wing, suggests that MaltOBP1 participates in other physiological activities in addition to perception and recognition of semiochemicals in *M. alternatus*. A number of OBPs were found to be expressed in non-olfactory tissues, including head (*R. ferrugineus* RferOBP7, Yan et al., 2016), thorax (*R. ferrugineus* RferOBP11, Yan et al., 2016), abdomen (*Agrilus mali* AmalOBP8, Cui et al., 2018), and palps (*C. capitata*, CcapOBP22, Falchetto et al., 2019), indicating that these OBPs play other physiological roles. For instance, orthologous OBP10 from two sibling *Helicoverpa* species was expressed in both chemosensory and reproductive organs, and a function as an oviposition deterrent in *Helicoverpa* species was suggested, potentially to avoid cannibalism of conspecific larvae (Sun et al., 2012). In *Drosophila melanogaster*, DmelOBP49a was expressed in gustatory organs, which are involved in the detection of bitter compounds to guide feeding decisions (Jeong et al., 2013). Higher expression in wing tissue was found in *A. mali* (Cui et al., 2018), *S. exigua* (Liu N. Y. et al., 2015), and *Ectropis oblique* (Ma et al., 2011). Cui et al. (2018) demonstrated the presence of chemoreceptors on the *A. mali* wing, suggesting that OBPs also play other physiological.

### Functional Analysis of MaltOBP1

MaltOBP1 has a specific binding preference for certain terpenoids, showing different affinities to (+)-α-pinene, β-pinene, (−)-*trans*-caryophyllene, (R)-(+)-limonene, and (−)-verbenone. Ligands with carbon chains are capable of conformational changes, altering their binding affinity. Previous studies showed that the length of the carbon chain in ligands played a key role in their binding affinity to proteins (Sun et al., 2016; Zheng et al., 2016). Here, we observed that MaltOBP1 showed the strongest binding affinity to (−)-*trans*-caryophyllene, in which C_15_H_24_, a natural sesquiterpene, has the longest carbon chain among the tested ligands, confirming that the length of the carbon chain in ligands is critical in shaping the binding affinity in *M. alternatus*. A previous study suggested that high concentrations of β-pinene could inhibit the consumption of bark-based diets by *M. alternatus* (Fan and Sun, 2006). The response of MalOBP1 to these host volatiles may involve elements of the olfactory system. MaltOBP3 and MaltOBP5 demonstrated high binding affinities to (R)-(+)-α-pinene, (+)-β-pinene, and (−)-limomene (Gao and Wang, 2014). Moreover, (R)-(+)-α-pinene and (+)-β-pinene were previously reported to be capable of eliciting strong electroantennogram (EAG) responses and to be efficient attractants for *M. alternatus* under both laboratory and field conditions (Fan et al., 2007a, b). Therefore, MaltOBP1 most likely participates in *M. alternatus* chemoreception of host plants, but further study is needed to confirm this. Among other MaltOBPs, MaltOBP9 shows high affinity with myrcene; MaltOBP10 with myrcene and (+)-α-pinene; MaltOBP19 with myrcene, (+)-α-pinene (+)-β-pinene, α-terpinolene and comphene; and MaltOBP24 with myrcene, (+)-3-carene and (+)-α-pinene (Wang, 2015), which means that different MaltOBPs may operate through different mechanisms in binding and releasing odorants.

In this study, MaltOBP1 showed no binding affinity with (+)-3-carene, myrcene, or comphene, which is consistent with the results from Lee et al. (2017, 2018). Previous studies demonstrated that the acidity of the surrounding environment could cause conformational changes in OBPs. For example, MaltOBP9 and MaltOBP13 showed higher binding affinity to (R)-(+)-limonene and α-terpinolene in acidic conditions, while no binding affinity was observed under the neutral conditions (Wang, 2015). Michel et al. (2011) suggested that pH-dependent conformational changes dictate the binding affinity of OBPs with odorants.

## Summary and Perspectives

In this study, we successfully cloned, expressed, and purified MaltOBP1 from *M. alternatus*. Immunoblot analysis indicated that MaltOBP1 is highly expressed in adult antennae, wings and legs. Binding assays further revealed that MaltOBP1 could selectively recognize host odorants. These results provide insight into the mechanism of olfactory recognition of *M. alternatus* and may assist in the development of new pest-prevention strategies for *M. alternatus*. In future studies, we will focus on the effects of the selected ligands on the behavior and ecology of *M. alternatus* in the context of longhorned beetle control. Furthermore, additional experiments, such as gene knockdown studies and site-directed mutagenesis, would be required for further verification of MaltOBP1’s physiological functions.

## Data Availability Statement

The datasets generated for this study can be found in the GenBank, accession numbers are listed in Supplementary Table S2.

## Author Contributions

XL and XZ conceived and designed the experiments. XL, ZZ, and FZ carried out the experiments. XL, FZ, and XZ analyzed the data. FZ, XL, YZ, QZ, and QW contributed reagents, materials, and analysis tools. FZ and XL drafted the manuscript. AM, JZ, and XZ provided editorial changes. All authors approved the final version of the manuscript.

## Conflict of Interest

The authors declare that the research was conducted in the absence of any commercial or financial relationships that could be construed as a potential conflict of interest.

## Footnotes

1 https://web.expasy.org/protparam/

2 https://www.cbs.dtu.dk/services/SignalP/

3 https://www.ncbi.nlm.nih.gov/

4 http://swissmodel.expasy.org/

5 http://www.pymol.org/

## Supplementary Material

FIGURE S1 MaltOBP1 antiserum titer tested by ELISA.

FIGURE S2 Nucleotide and deduced amino acid sequences of MaltOBP1. The predicted signal peptide at the N-terminus is underlined, the four conserved cysteines are boxed, and the stop codon is indicated with an asterisk.

FIGURE S3 Sequence alignment of 12 homologous Minus-C OBPs. The letters in the frame represent four conserved cysteines in the Minus-C OBP. Malt_OBP1 (*Monochamus alternates*): ABR53888.1; Malt_OBP12: AIX97027.1; Malt_OBP22: AIX97037.1; Malt_OBP23: AIX97038.1; Achi_OBP (*Anoplophora chinensis*): AUF72967.1; Agla_OBP1 (*Anoplophora glabripennis*): ATG83411.1; Agla_OBP72: XP_018563348.1; Agla_OBP83a: XP_018563349.1; Bhor_OBP2 (*Batocera horsfieldi*):; Ldec_OBP83a (*Leptinotarsa decemlineata*): XP_023027761.1; Rfer_OBP10 (*Rhynchophorus ferrugineus*): ANE37554.1; Rfer_OBP107: AVR54529.1; Xqua_OBP1 (*Xylotrechus quadripes*): AXO78379.1.

FIGURE S4 Phylogenetic analysis of MaltOBP1 with other Coleopteran insects. MaltOBP1 is indicated in the arrows. The closest relative OBPs with MaltOBPs are highlighted with light pink color. The protein names and sequences of the 91 OBPs that were used included OBPs from *Monochamus alternatus* (Malt), *Anoplophora glabripennis* (Agla), *Tribolium castaneum* (Tcas), and *Leptinotarsa decemlineata* (Ldec). The accession numbers of genes used to construct phylogenetic tree are listed in Supplementary Table S2, and the genes from *L. decemlineata* refer to Liu Y. et al. (2015) and Zhang et al. (2019).

FIGURE S5 Analysis of the size and purity of recombinant MaltOBP1 protein by LC-MS.

TABLE S1 Primers used in this study.

TABLE S2 The accession numbers of genes used to construct phylogenetic tree in this study.
