# Supplementary figures and images for: Characterization of MaltOBP1, a Minus-C Odorant-Binding Protein, From the Japanese Pine Sawyer Beetle, Monochamus alternatus Hope (Coleoptera: Cerambycidae)
Source: Front Physiol. 2020 Apr 1;11:212. doi: 10.3389/fphys.2020.00212 (PMC7138900; doi:10.3389/fphys.2020.00212)

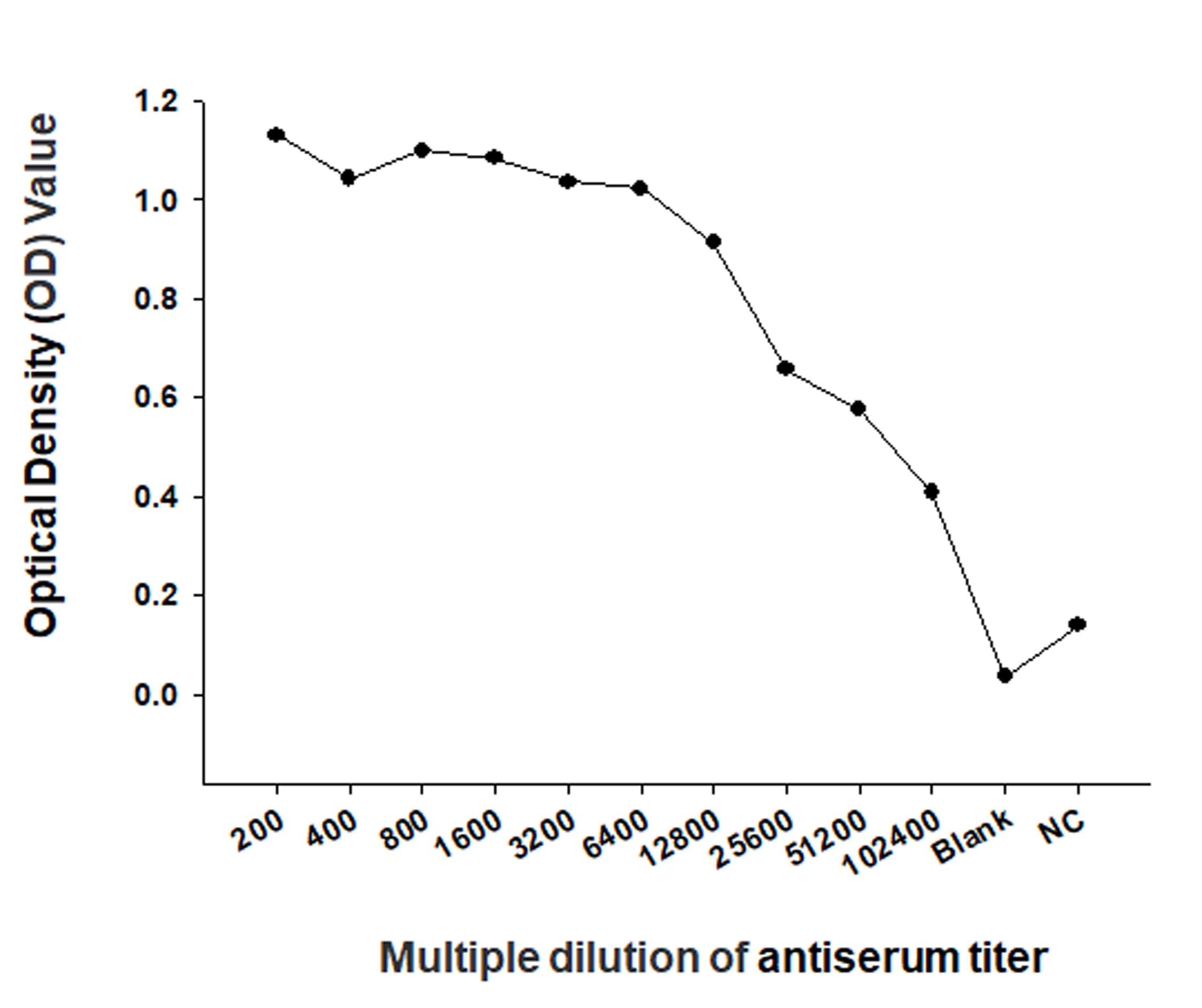

Supplement: FIGURE S1 — MaltOBP1 antiserum titer tested by ELISA. [file Image_1.TIF]

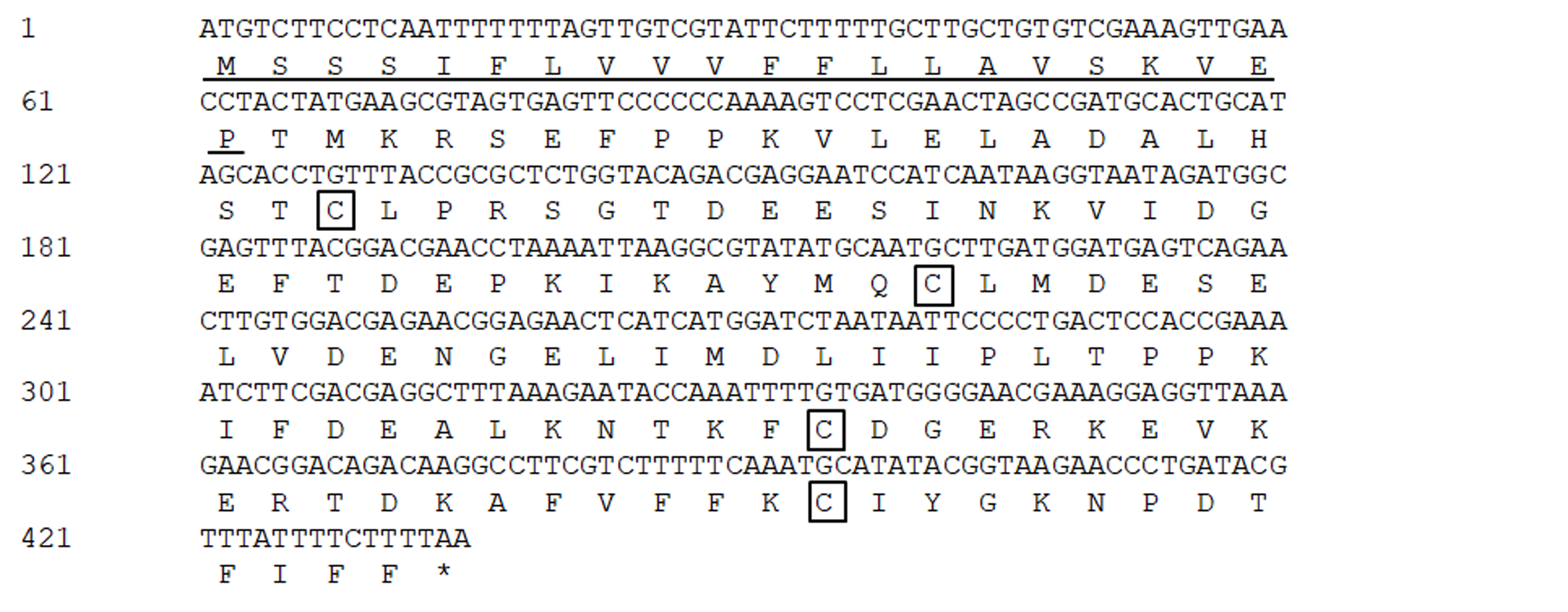

Supplement: FIGURE S2 — Nucleotide and deduced amino acid sequences of MaltOBP1. The predicted signal peptide at the N-terminus is underlined, the four conserved cysteines are boxed, and the stop codon is indicated with an asterisk. [file Image_2.TIF]

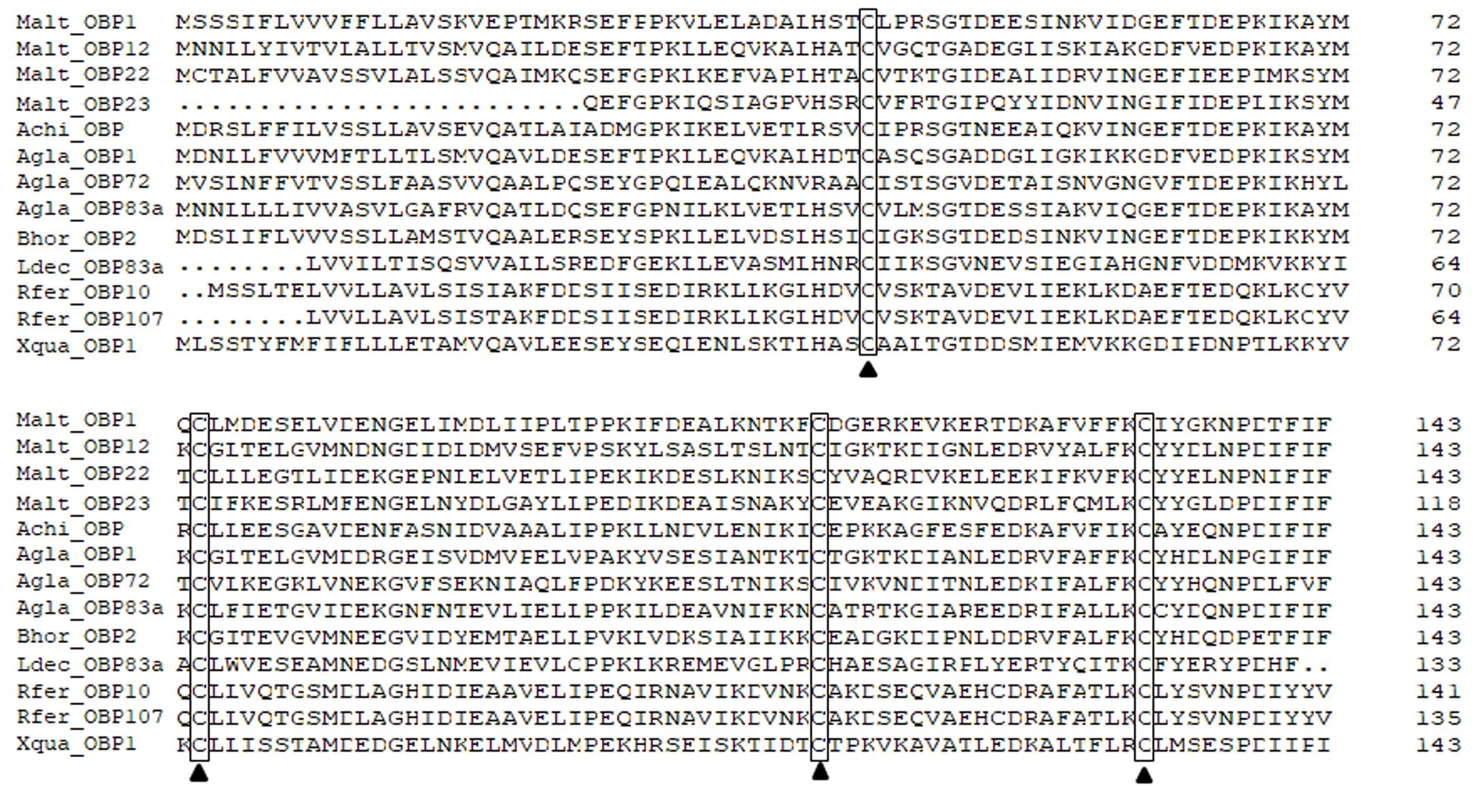

Supplement: FIGURE S3 — Sequence alignment of 12 homologous Minus-C OBPs. The letters in the frame represent four conserved cysteines in the Minus-C OBP. Malt_OBP1 (Monochamus alternates): ABR53888.1; Malt_OBP12: AIX97027.1; Malt_OBP22: AIX97037.1; Malt_OBP23: AIX97038.1; Achi_OBP (Anoplophora chinensis): AUF72967.1; Agla_OBP1 (Anoplophora glabripennis): ATG83411.1; Agla_OBP72: XP_018563348.1; Agla_OBP83a: XP_018563349.1; Bhor_OBP2 (Batocera horsfieldi):; Ldec_OBP83a (Leptinotarsa decemlineata): XP_023027761.1; Rfer_OBP10 (Rhynchophorus ferrugineus): ANE37554.1; Rfer_OBP107: AVR54529.1; Xqua_OBP1 (Xylotrechus quadripes): AXO78379.1. [file Image_3.TIF]

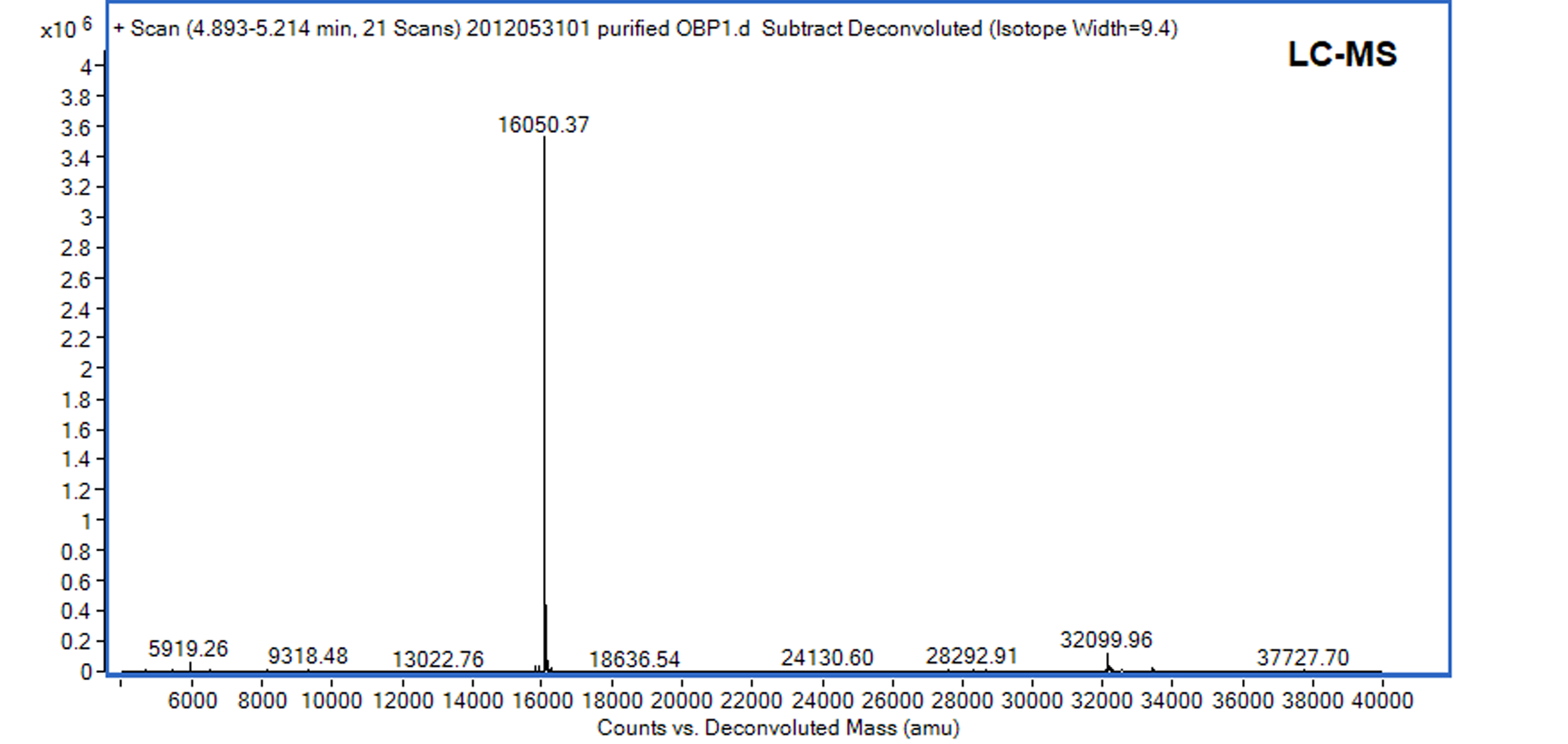

Supplement: FIGURE S4 — Phylogenetic analysis of MaltOBP1 with other Coleopteran insects. MaltOBP1 is indicated in the arrows. The closest relative OBPs with MaltOBPs are highlighted with light pink color. The protein names and sequences of the 91 OBPs that were used included OBPs from Monochamus alternatus (Malt), Anoplophora glabripennis (Agla), Tribolium castaneum (Tcas), and Leptinotarsa decemlineata (Ldec). The accession numbers of genes used to construct phylogenetic tree are listed in Supplementary Table S2, and the genes from L. decemlineata refer to Liu Y. et al. (2015) and Zhang et al. (2019). [file Image_4.TIF]

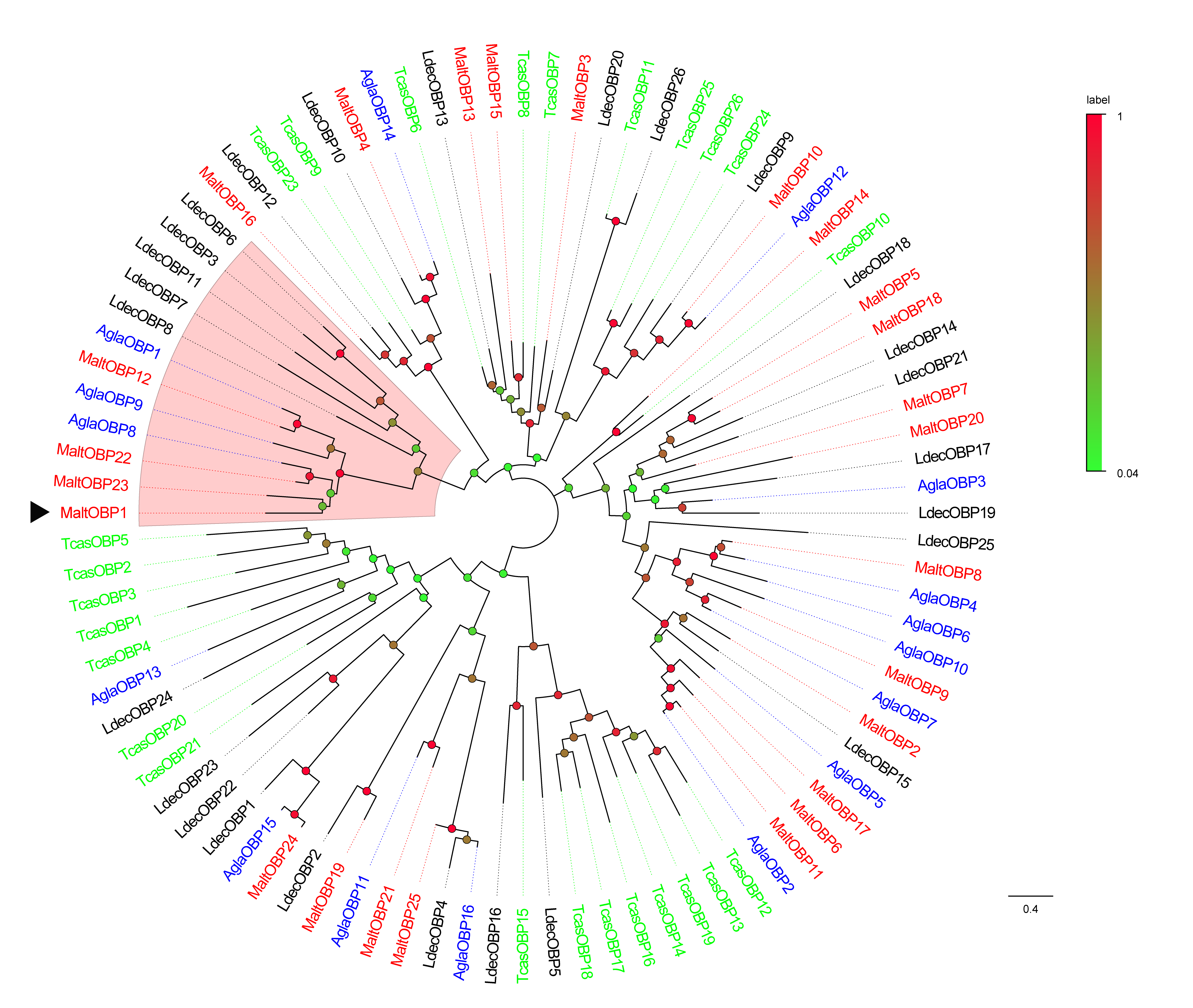

Supplement: FIGURE S5 — Analysis of the size and purity of recombinant MaltOBP1 protein by LC-MS. [file Image_5.TIF]
