# Supplementary material for: Characterization of MaltOBP1, a Minus-C Odorant-Binding Protein, From the Japanese Pine Sawyer Beetle, Monochamus alternatus Hope (Coleoptera: Cerambycidae)
Source: Front Physiol. 2020 Apr 1;11:212. doi: 10.3389/fphys.2020.00212 (PMC7138900; doi:10.3389/fphys.2020.00212)
Supplement: TABLE S1 — Primers used in this study. [file Table_1.docx]

**Supplementary Materials**

**Table S1. Primers used in this study**

| **Purpose** | **Primer name** | **Primer sequence (5’-3’)** |
| --- | --- | --- |
| **The first strand of cDNA** | Oligo dT-3sites adaptor primer | GGCCACGCGTCGACTAGTACTTTTTTTTTTTTTTTTTTVN |
| **3′ RACE** | Malt-OBP 3’ Primer 1 (sense) | ATHAARGAYGARTCNGA |
|  | Malt-OBP 3’ Primer 2 (sense) | ATHAARGAYGARAGYGA |
|  | Adapter Primer (antisense) | CTGATCTAGAGGTACCGGATCC |
| **5′ RACE** | GSP Primer (antisense) | TAGGTGGAGTCAACGGGATTAT |
|  | Outer Primer (sense) | GCTGATGGCGATGAATGAACACTG |
|  | Inner Primer (sense) | CGCGGATCCGAACACTGCGTTTGCTGGCTTTGATG |
| **Internal reference** | Actin forward primer | CAAGCAAGAATACGACGAATCCG |
|  | Actin reverse primer | CAGTACAACCACGGTCGCATTAA |
| **Recombinant protein** | pET32a_N-His_TEV_F | GCCCTGAAAATACAGGTTTTCAGAAGAATGATGATGATGATGGTGCA |
|  | pET32a_N-His_TEV_R | TGAGATCCGGCTGCTAACAAA |
|  | pET32a_N-His_TEV_OBP1_T1F | GAAAACCTGTATTTTCAGGGCACCATGAAACGTAGCGAATTTC |
|  | pET32a_N-His_TEV_OBP1_F123R | TTTGTTAGCAGCCGGATCTCAGAAGAAGATGAAGGTGTCC |

**Table S2.** **Genes used to construct the phylogenetic tree in this study**

| **OBPs** | **Accession #*** | **OBPs** | **Accession #** | **OBPs** | **Accession #** |
| --- | --- | --- | --- | --- | --- |
| MaltOBP1 | ABR53888.1 | TcasOBP1 | EFA05678.1 | AglaOBP1 | ARU83752.1 |
| MaltOBP2 | AHA39267.1 | TcasOBP2 | EFA05676.2 | AglaOBP2 | ARU83753.1 |
| MaltOBP3 | AHA39268.1 | TcasOBP3 | EFA05675.1 | AglaOBP3 | ARU83754.1 |
| MaltOBP4 | AHA39269.1 | TcasOBP4 | EFA05742.1 | AglaOBP4 | ARH65459.1 |
| MaltOBP5 | AHA39270.1 | TcasOBP5 | EFA05677.1 | AglaOBP5 | ARH65460.1 |
| MaltOBP6 | AJO67868.1 | TcasOBP6 | EFA04594.1 | AglaOBP6 | ARH65461.1 |
| MaltOBP7 | AIX97022.1 | TcasOBP7 | EFA04593.1 | AglaOBP7 | ARH65462.1 |
| MaltOBP8 | AIX97023.1 | TcasOBP8 | EFA04687.2 | AglaOBP8 | ARH65463.1 |
| MaltOBP9 | AIX97024.1 | TcasOBP9 | EFA10713.1 | AglaOBP9 | ARH65464.1 |
| MaltOBP10 | AIX97025.1 | TcasOBP10 | EFA07542.1 | AglaOBP10 | ARH65465.1 |
| MaltOBP11 | AIX97026.1 | TcasOBP11 | EFA05695.1 | AglaOBP11 | ARH65466.1 |
| MaltOBP12 | AIX97027.1 | TcasOBP12 | EFA02857.1 | AglaOBP12 | ARH65467.1 |
| MaltOBP13 | AIX97028.1 | TcasOBP13 | EFA02858.1 | AglaOBP13 | ARH65468.1 |
| MaltOBP14 | AIX97029.1 | TcasOBP14 | EFA02914.1 | AglaOBP14 | ARH65469.1 |
| MaltOBP15 | AIX97030.1 | TcasOBP15 | EFA12066.1 | AglaOBP15 | ARH65470.1 |
| MaltOBP16 | AIX97031.1 | TcasOBP16 | EFA02853.2 | AglaOBP16 | ARH65471.1 |
| MaltOBP17 | AIX97032.1 | TcasOBP17 | EFA02861.1 |  |  |
| MaltOBP18 | AIX97033.1 | TcasOBP18 | EFA02860.1 |  |  |
| MaltOBP19 | AIX97034.1 | TcasOBP19 | EFA02960.1 |  |  |
| MaltOBP20 | AIX97035.1 | TcasOBP20 | EFA05793.2 |  |  |
| MaltOBP21 | AIX97036.1 | TcasOBP21 | EFA09215.2 |  |  |
| MaltOBP22 | AIX97037.1 | TcasOBP23 | EFA10803.1 |  |  |
| MaltOBP23 | AIX97038.1 | TcasOBP24 | EFA04576.1 |  |  |
| MaltOBP24 | AIX97039.1 | TcasOBP25 | EFA04747.2 |  |  |
| MaltOBP25 | AIX97019.1 | TcasOBP26 | EFA04746.2 |  |  |

“*”: Accession number
